# Multidrug-resistant HIV viral rebound during early syphilis: a case report

**DOI:** 10.1186/s12879-020-04999-4

**Published:** 2020-04-07

**Authors:** Andrea Giacomelli, Valeria Micheli, Dario Cattaneo, Alessandro Mancon, Cristina Gervasoni

**Affiliations:** 1 Department of Biomedical and Clinical Sciences, DIBIC Luigi Sacco, Milan University, Via G.B. Grassi 74, 20157 Milan, Italy; 2 III Infectious Disease Unit, ASST Fatebenefratelli Sacco University Hospital, Milan, Italy; 3 Clinical Microbiology, Virology and Bioemergencies, ASST Fatebenefratelli Sacco University Hospital, Milan, Italy; 4 Gestione Ambulatoriale Politerapie (GAP) Outpatient Clinic, ASST Fatebenefratelli Sacco University Hospital, Milan, Italy; 5 Unit of Clinical Pharmacology, ASST Fatebenefratelli Sacco University Hospital, Milan, Italy

**Keywords:** Syphilis, Transmitted drug resistance, Virological rebound, HIV prevention, Therapeutic drug monitoring, Case report

## Abstract

**Background:**

Syphilis has been associated with an increase in HIV RNA and a temporary decline in CD4 T cell counts in people living with HIV who are not receiving antiretroviral treatment (ART), and may be associated with a transient HIV RNA rebound in those who are receiving ART. Our case is the first to highlight the risk of a multidrug-resistant HIV viral rebound during the course of early syphilis even if antiretroviral drug concentrations are within the therapeutic range.

**Case presentation:**

This 50-year-old HIV-1-positive male patient with concomitant early syphilis presented with an HIV RNA rebound (8908 copies/mL) during a scheduled visit to our clinic. He was receiving a stable ART regimen consisting of darunavir/cobicistat plus dolutegravir, and had a 15-year history of viral suppression. Good short-term drug adherence could be inferred as liquid chromatography tandem mass spectrometry showed that his trough antiretroviral drug concentrations were within the therapeutic range: darunavir 2353 ng/mL (minimum effective concentration > 500 ng/mL) and dolutegravir 986 ng/mL (minimum effective concentration > 100 ng/mL). A plasma RNA genotype resistance test revealed wild-type virus in the integrase region and protease region (PR), but extensive resistance in the reverse transcriptase (RT) region (M41L, E44D, D67N, K70R, M184V, L210W and T215Y). Phylogenetic analysis of next-generation sequences (used to investigate the presence of minor viral variants), the PR and RT sequences from plasma HIV RNA and pro-viral DNA extracted from peripheral blood mononuclear cells during the viral rebound, and a Sanger sequence obtained during a previous virological failure suggested clonal viral expression because the previous PR resistance mutations had been lost or had not been archived in pro-viral DNA.

**Conclusions:**

This case shows that early syphilis may cause an HIV RNA rebound in patients under stable virological control with the potential of transmitting an extensively drug-resistant virus.

## Background

There has recently been an increase in the incidence of sexually transmitted diseases (STDs) among men who have sex with men (MSM), whether they are people living with HIV/AIDS (PLWHAs) or the recipients of pre-exposure prophylaxis (PrEP) [1, 2]. Syphilis is an STD that has been associated with an increase in HIV RNA and a temporary decline in cluster of differentiation (CD) 4 T cell counts in patients not receiving antiretroviral treatment (ART) [3], and concomitant early syphilis has been associated with a transient rebound in HIV viral load in PLWHAs receiving effective ART [4].

We here describe the case of an HIV-infected MSM with early syphilis experiencing a viral rebound and showing extensive resistance in the reverse transcriptase (RT) region. This is the first case to highlight the risk of an HIV multidrug-resistant viral rebound during early syphilis even when antiretroviral drug concentrations are within the therapeutic range.

## Case presentation

**Fig. 1:**
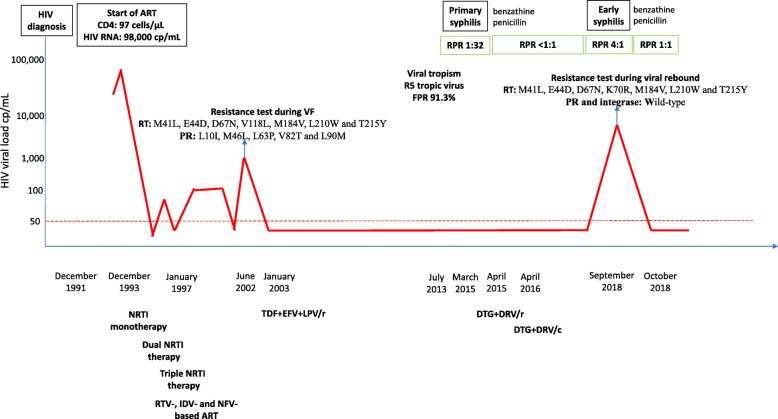
The patient’s medical history. The continuous red line represents the trend of HIV RNA levels over time, and the dashed red line represents the threshold of HIV RNA levels of < 50 copies/μL. The boxes contain brief descriptions of major clinical events. A schematic representation of the patient’s history of ART is given below the graph. Abbreviations: cp: copies; ART: antiretroviral treatment; VF: virological failure; RPR: rapid plasma reagin; FPR: false positive rate; RT: reverse transcriptase; PR: protease; NRTIs: nucleoside reverse transcriptase inhibitors; RTV: ritonavir; IDV: indinavir; NFV: nelfinavir; TDF: tenofovir; EFV: efavirenz; LPV/r: lopinavir/ritonavir; DTG: dolutegravir; DRV/r: darunavir/ritonavir; DRV/c: darunavir/cobicistat

During a scheduled visit in September 2018, a 50-year-old MSM with early syphilis infection, who had tested positive for clade B HIV-1 in December 1991 and had subsequently been regularly followed up at our centre, was found to be affected by an HIV RNA rebound (8908 copies/mL). Figure 1 shows the patient’s medical history: he started ART in December 1993 with a CD4 T cell nadir of 97 cells/μL and an HIV RNA zenith of 98,000 copies/mL. It is worth noting that he initially received sub-optimal ART based on a combination of thymidine analogues, nucleoside RT inhibitors, and the first protease (PR) inhibitors that led to multiple virological failures (VFs). A genotype resistance test (GRT) in June 2002 (Trugene® HIV-1 Genotyping Assay, Siemens Healthcare Diagnostics GmbH, Eschborn, Germany) showed extensive resistance in the RT region (M41L, E44D, D67N, V118L, M184V, L210W, and T215Y) and the PR region (L10I, M46L, L63P, V82T, and L90M), and a tropism test in 2013 (envelope glycoprotein (gp)120 V3 loop sequencing) revealed a C-C chemokine receptor type 5 (CCR5) tropic virus with a false positive rate of 91.3% (geno2pheno co-receptor tool). However, the patient had experienced stable virological suppression from 2003, when he started an ART regimen based on tenofovir disoproxil fumarate (TDF), efavirenz (EFV), and lopinavir/ritonavir (LPV/r), which was simplified to darunavir/ritonavir (DRV/r) and dolutegravir (DTG) in April 2015, and further simplified to DRV/cobicistat (DRV/c) and DTG in April 2016.

At the time of the viral rebound, the patient’s syphilis was considered to be in an early stage as a rapid plasma reagin (RPR) test within 12 months of the index event was negative after treatment with benzathine penicillin for primary syphilis infection (RPR titre down from 1:32 to < 1:1 in March 2015), but the titre had increased to 1:4 in December 2017. Moreover, the patient reported that he had had multiple condomless, insertive/receptive anal intercourses in the previous months.

After being purified by means of solid-phase extraction, the plasma sample used to quantify his HIV RNA viral load (handled on ice and stored at − 20 °C) was used to determine trough antiretroviral drug concentrations by means of liquid chromatography tandem mass spectrometry. The results indicated good short-term adherence to the prescribed ART as the trough concentrations were within the therapeutic range (DRV 2353 ng/mL, minimum effective concentration > 500 ng/mL; DTG 986 ng/mL, minimum effective concentration > 100 ng/mL). Furthermore, all of the previous assessments of the trough concentrations of both drugs (carried out at least twice a year in our clinical practice) had always shown levels within the therapeutic range, which provides indirect evidence of long-term treatment compliance.

**Fig. 2:**
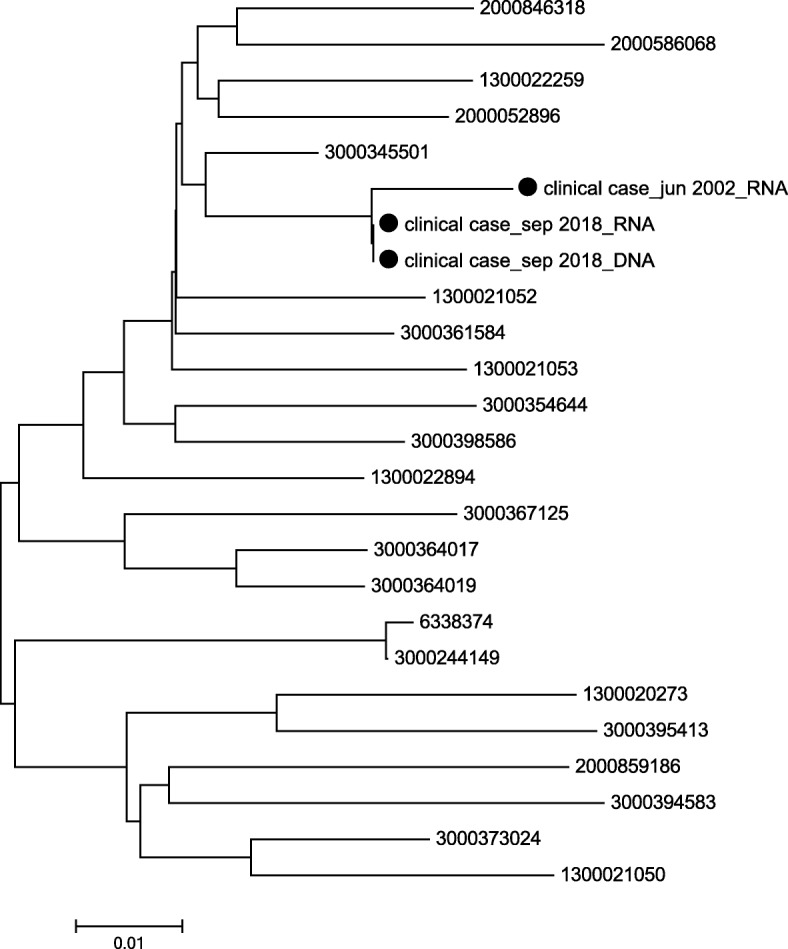
Phylogenetic tree showing the relationships between the three PR and RT Sanger sequences collected from the patient during virological failure in June 2003 (HIV RNA) and during the virological rebound concomitant with early syphilis in September 2018 (HIV RNA and HIV DNA)

Viral rebound was assessed by means of genotype resistance tests (GRTs, Viroseq HIV-1 Genotyping System v. 2, ViroSeq; Abbott GmbH, Wiesbaden, Germany). The test of plasma RNA showed wild-type virus in the PR and integrase region, but extensive resistances in the RT region (M41L, E44D, D67N, K70R, M184V, L210W and T215Y), and the test of plasma DNA showed the same resistance profile. In order to investigate the presence of minor viral variants, next-generation sequencing analysis (NGS) of the same samples (NGS HIV-1 Solution kit, Arrow Diagnostics S.r.l., Genova, Italy) revealed the presence of PR variants in < 1.0% of the viral population in the plasma RNA sample (DNA data are not available because of a failure in the amplification process). Phylogenetic analyses of Sanger PR and RT sequences obtained from plasma HIV RNA and pro-viral DNA extracted from peripheral blood mononuclear cells during the viral rebound, and a sequence obtained from the same patient during a previous VF in 2002 revealed close correlations, especially between the RNA and DNA samples simultaneously collected in 2018 (Fig. 2). These findings suggested clonal viral expression as previous PR resistance mutations had been lost or not archived in the pro-viral DNA.

Four weeks after the viral rebound, the patient was still on the same ART with undetectable HIV RNA and, 3 months after treatment with benzathine penicillin, the RPR titre was 1:1.

## Discussion and conclusions

The finding that there is no viral transmission between sero-different couples when the HIV-positive partner has HIV RNA levels below the threshold of 200 copies/mL [5] has led to the undetectable = untransmittable (U=U) campaign [6], which has reduced the stigma associated with HIV infection and promoted the retention in care of PLWHAs [7]. It is also well known that factors such as bacterial infections, influenza vaccinations and poor adherence to ART can cause a transient viral rebound that may not require a change in antiretroviral regimen: interestingly, Kolber et al. found that seven of their 34 patients experienced a viral rebound after an influenza vaccination, two of whom showed RT and PR mutations [8–11], although it is not clear whether these were primary mutations or a spillover of previous archived mutations [10].

It has been reported that the incidence of syphilis (like that of other STDs) is increasing among MSM worldwide [1, 2], and a number of authors have postulated that early syphilis increases HIV viremia and reduces CD4 T cell counts [3, 4]. The first study of a mixed populations of patients with uncontrolled HIV viremia and patients receiving ART found that the patients with HIV RNA levels of < 500 copies/mL and early syphilis experienced an increased viral load during the first 6 months after the presumed date of infection: 27% of the patients with early syphilis showed an increase in HIV RNA to between 1392 and 10,500 copies/mL, with a median duration of detectable viremia of 1.6 months (interquartile range: 1.6–7.3), and the authors suggested that early syphilis increased the risk of HIV transmission even in patients with HIV RNA levels of < 500 copies/mL [4].

Conversely, Grewal et al. did not observe an increased risk of VF in a Canadian cohort of PLWHA MSM with acute syphilis who were receiving effective ART: the overall incidence of VF was 3.5/100 person-years (95% confidence interval: 3.4–4.2) and, as there was no statistically significant association with acute syphilis, the authors concluded that ART may still reduce the risk of transmitting HIV to sexual partners [12]. However, this conclusion is undermined by the fact that it is questionable whether syphilis causes VF or simply a temporary loss of virological control that can often be regained without changing ART. Secondly, the short duration of detectable HIV viremia following an episode of early syphilis in patients under virological control [4] suggests that a virological rebound may be limited between two consecutive HIV RNA determinations.

Our phylogenetic analysis reasonably excluded the possibility that the mutations observed during our patient’s viral rebound were due to HIV super-infection [13, 14], but the rebound may have been due to previously archived viruses. Interestingly, the RT mutations were similar to those found during a previous VF, but the previously detected PR mutations were not identified. This may be partially explained by the different dynamics of HIV resistance mutations in the absence of antiretroviral pressure: the loss of RT mutations is slower than the loss of PR mutations [15].

At the time of the viral rebound, our patent had trough concentrations of antiretrovirals within the therapeutic range. As therapeutic drug monitoring (TDM) can only provide evidence of short-term compliance to treatment, we cannot exclude the possibility that he may not have been compliant in the weeks before the event. However, he was regularly followed up at our clinic and all of the previous TDM assessments (made at least twice a year) also showed therapeutic drug concentrations, thus indirectly suggesting long-term adherence.

Our case suggests that early syphilis can cause an albeit time-limited rebound of HIV viremia in patients with self reported good adherence to ART and antiretroviral trough concentrations within the therapeutic range. The HIV RNA levels reached during the rebound are consistent with a high rate of HIV transmission [16], and the highly resistant nature of the detected virus is worth noting because of the potential risk of transmitting a drug-resistant virus. The vigilant monitoring of STDs, particularly syphilis, in such a high-risk population is necessary in order to be able to ensure an early diagnosis and prompt treatment.

## Data Availability

Data will be made available upon reasonable request.

## Abbreviations

STDs: Sexual transmitted diseases
MSM: Men who have sex with men
PLWHA: People living with HIV/AIDS
PrEP: Pre exposure prophylaxis
ART: Antiretroviral treatment
CD: Cluster of differentiation
RT: Reverse transcriptase
RPR: Rapid plasma reagin
GRT: Genotype resistance test
CCR5: C-C chemokine receptor type 5
gp: glycoprotein
NGS: Next-generation sequencing analysis
PR: Protease
VF: Virological failure
DRV: Darunavir
DTG: Dolutegravir
TDF: Tenofovir disoproxil fumarate
EFV: Efavirenz
LPV/r: Lopinavir/ritonavir
DRV/r: Darunavir/ritonavir
DRV/c: Darunavir/cobicistat
U=U: Undetectable = untransmittable
TDM: Therapeutic drug monitoring
